# The Outbreak of Typhoid at University College Hospital

**Published:** 1897-10-16

**Authors:** 


### THE OUTBREAK OF TYPHOID AT UNIVERSITY COLLEGE HOSPITAL.

The occurrence of a number of cases of typhoid fever among the nurses and servants at University College Hospital is very unfortunate, in that it suggests to the lay mind many erroneous ideas as to the infectiousness of the disease. In this instance the outbreak has all the characteristics of what is somewhat crudely spoken of as "milk typhoid." The question where the milk became contaminated, and how far, if at all, any connection can be traced between the cases of fever usually present in the ward and the arrangements made for the storage of milk or other foods, is a matter of much interest. What is certain, however, is that in this instance the disease has not been caught while nursing, and is not due to any implication of the general water supply.

